# Distinct neural mechanisms underlying the effects of agility and resistance-aerobic training on executive function and gait in young adults

**DOI:** 10.3389/fpsyg.2026.1743946

**Published:** 2026-06-10

**Authors:** Shiuan-Ling Huang, Yan-Ci Liu

**Affiliations:** 1 School and Graduate Institute of Physical Therapy, College of Medicine, National Taiwan University, Taipei, Taiwan; 2 Department of Rehabilitation, Taipei Tzu Chi Hospital, Buddhist Tzu Chi Medical Foundation, New Taipei City, Taiwan; 3 Physical Therapy Center, National Taiwan University Hospital, Taipei, Taiwan

**Keywords:** agility training, brain activity, dual task walking, executive function, resistance-aerobic training

## Abstract

**Introduction:**

Executive function (EF) is essential for daily functioning. While exercise enhances EF, the specific effects of different training types remain unclear. Agility training (AT), which integrates cognitive and motor demands, is a promising but understudied intervention. This study compared the effects of AT versus combined resistance-aerobic training (RAeT) on EF, dual-task walking, and their underlying neural mechanisms in healthy adults.

**Methods:**

In this randomized controlled trial, 26 healthy adults were assigned to either AT (*n* = 13) or RAeT (*n* = 13) group. Both interventions consisted of nine 50-min sessions over 3 weeks. Assessments were conducted at pre-intervention, post-intervention, and one-month follow-up, measuring EF (working memory, inhibitory control, and cognitive flexibility), dual-task walking, and brain activation via functional near-infrared spectroscopy (fNIRS).

**Results:**

AT and RAeT induced distinct behavioral and neural patterns. AT primarily enhanced executive function, significantly reducing Stroop incongruent response times (Pre: 1085.20 ms; Post: 907.96 ms; F/u: 843.00 ms; Pre-Post *p* = 0.016, Pre-F/u *p* = 0.002). Conversely, RAeT significantly improved cognitive processing speed during dual-task walking (Pre: 0.26/s; Post: 0.29/s; F/u: 0.28/s; Pre-Post *p* = 0.018, Pre-F/u *p* = 0.008). These behavioral changes were mirrored by different neural patterns: during EF tasks, the AT group exhibited lower brain activation in prefrontal and premotor cortices, suggesting a more economical neural recruitment pattern. During dual-task walking, the RAeT group also demonstrated lower brain activation, consistent with enhanced motor automaticity.

**Conclusion:**

These preliminary findings suggest that AT and RAeT may improve cognitive-motor function through distinct neurophysiological mechanisms. AT optimized efficiency in reactive cognitive control, while RAeT enhanced efficiency in integrated cognitive-motor activities. Agility training is thus a flexible, practical, and cost-effective option for improving brain and cognitive function, especially in settings with limited equipment.

## 1 Introduction

Executive function (EF) refers to a set of high-level cognitive processes essential for goal-directed behavior, including the regulation of attention, inhibition, working memory, and cognitive flexibility (Yogev-Seligmann et al., 2008; Diamond, 2013; Chen et al., 2014; Liu-Ambrose et al., 2008). These core domains enable individuals to plan, initiate, monitor, and adapt their actions, and are closely linked to real-life functioning such as multitasking, problem solving, and decision making (Diamond, 2013; Chen et al., 2014; Liu-Ambrose et al., 2008). EF also plays a crucial role in the coordination between sensory input and behavioral output, making it a key component in cognitive-motor control (Yogev-Seligmann et al., 2008).

Physical exercise has been shown to positively influence EF (Barha et al., 2017; Constans et al., 2016; Northey et al., 2018), potentially through various physiological mechanisms. Aerobic exercise stimulates neurotrophic factors such as brain-derived neurotrophic factor (BDNF) and insulin-like growth factor-1 (IGF-1), promoting synaptic plasticity and neurogenesis, while resistance exercise activates IGF-1 and AKT signaling pathways (Barha et al., 2017). Human studies have demonstrated that aerobic training improves EF and long-term memory (Northey et al., 2018), while resistance training enhances information processing speed, attention, and working memory (Constans et al., 2016). Combining aerobic with resistance training may provide synergistic benefits for cognitive and motor functions (Constans et al., 2016; Northey et al., 2018).

Recently, agility training has gained increasing attention as a multidimensional intervention that integrates physical and cognitive demands (Lennemann et al., 2013; Holmberg, 2009; Turner, 2011). Agility is defined as the ability to rapidly change direction in response to external stimuli, requiring not only physical skills such as strength and coordination, but also perceptual and decision-making abilities (Turner, 2011). Training typically progresses from technical drills and pattern running to reactive agility training and temporal occlusion training, incorporating varying degrees of cognitive challenge (Turner, 2011). These components parallel the demands of EF, particularly in terms of inhibition, working memory, and cognitive flexibility (Diamond, 2013; Chen et al., 2014; Liu-Ambrose et al., 2008), suggesting agility training may serve as an efficient alternative or complement to conventional exercise paradigms in promoting cognitive performance.

Despite these theoretical advantages, few studies have explored the cognitive effects of agility training. Morat et al. (2020) proposed an agility protocol targeting spatial orientation, balance, and strength, and recommended evaluating inhibitory control and working memory in older adults. Lennemann et al. (2013) reported improved working memory and attention in military personnel after agility training. Lichtenstein et al. (2020) also demonstrated motor improvements in older adults following agility training, though no cognitive outcomes were assessed. Thus, although preliminary findings suggest cognitive and neuromuscular benefits, the underlying mechanisms of agility training on EF remain poorly understood.

In addition to cognitive testing, dual-task walking has emerged as a sensitive and ecologically valid method to assess the interaction between cognition and motor control. This paradigm requires individuals to perform a cognitive task while walking, thereby engaging executive resources such as divided attention and working memory (Yogev-Seligmann et al., 2008; Holtzer et al., 2011). Improvements in dual-task gait performance may reflect enhanced EF and functional integration (Liao et al., 2019).

To investigate the neurophysiological basis of these cognitive and motor changes, functional near-infrared spectroscopy (fNIRS) was employed. fNIRS non-invasively measures cortical activation through hemoglobin changes during cognitive and motor tasks, offering real-time brain monitoring suitable for physical activity studies (Liu et al., 2018; Leff et al., 2011; Vitorio et al., 2017). Previous fNIRS research suggests cortical activation patterns may indicate neural efficiency or compensatory mechanisms depending on training type and cognitive demand (Vitorio et al., 2017; Guo et al., 2017; Al-Yahya et al., 2018).

Therefore, this study aimed to examine the effects of agility training versus combined resistance-aerobic training on EF, dual-task walking, and cortical activation in healthy adults. The study hypothesized that agility training is another economical and effective way as resistance combined aerobic training to improving cognitive and motor function in healthy adults through distinct neurophysiological pathways.

## 2 Materials and methods

### 2.1 Study design

**Figure 1:**
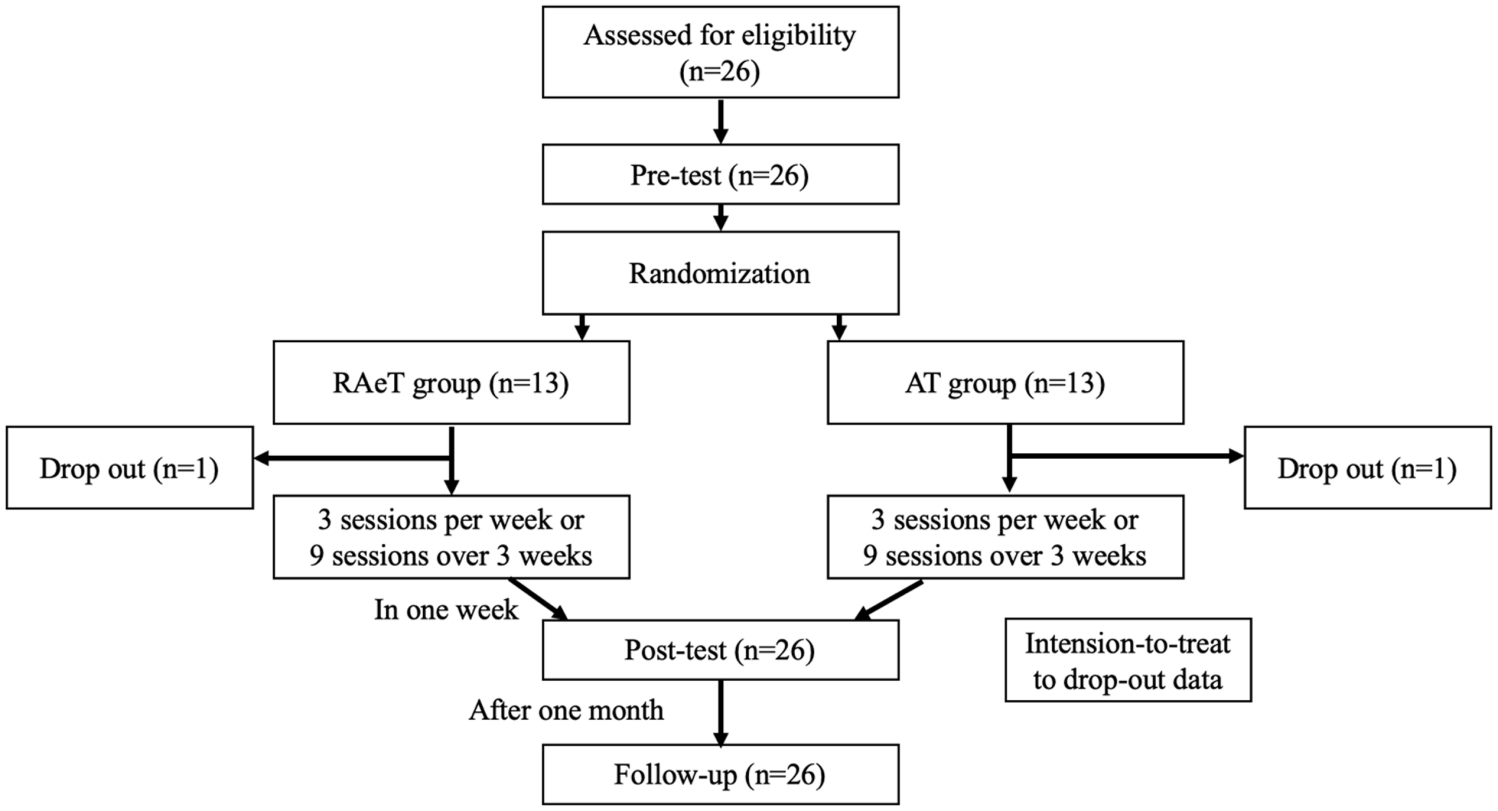
Flow chart of this study. RAeT, resistance + aerobic training; AT, agility training.

This was a randomized controlled trial with three assessments: pre-intervention, post-intervention, and 1-month follow-up. Participants were randomly allocated to either agility training (AT) or combined resistance-aerobic training (RAeT) group. The random allocation sequence was generated using a computer random number generator. Allocation concealment was maintained using sequentially numbered, opaque, sealed envelopes prepared in advance. Due to the nature of the exercise intervention, participants and exercise trainers could not be blinded. Both groups underwent nine training sessions (3 sessions/week) over 3 weeks, followed by post-testing and a follow-up assessment 1 month later (Figure 1). Regarding adherence, 24 of the 26 randomized participants (92.3%) completed the full 9-session training protocol. Two participants (one from each group) withdrew after the pre-test due to personal reasons. To maintain the integrity of the randomized groups, their data were analyzed following the intention-to-treat (ITT) principle. This study was registered at ClinicalTrials.gov (NCT04880057; initial release: 05/03/2021). The trial protocol and statistical analysis plan were predefined and registered prior to trial commencement, and no significant changes were made thereafter.

### 2.2 Participation

A total of 26 healthy adults participated in the study. Inclusion criteria were (1) age between 20 to 30 years, (2) ability to walk 20 meters independently without assistive devices, and (3) no known neuromuscular or cardiopulmonary diseases affecting exercise performance. The participants who were (1) overweight (BMI > 24), (2) taking medicine affecting cerebral circulation, and (3) any neurologic disorders, smoker, or alcohol abusers had been excluded. Participants were instructed to maintain their consistent daily routines, including dietary habits, sleep, and physical activity, throughout the study. All participants provided written informed consent prior to participation. The research was conducted in compliance with the principles outlined in the Declaration of Helsinki, and the study received approval from the Ethical Review Board at National Taiwan University Hospital (202103055RINB). Patients or members of the public were not involved in the design, conduct, reporting, or dissemination plans of our research.

### 2.3 Intervention

Both intervention groups involved group-based exercise sessions, with containing 3–5 persons per group. There was 50 min each session which is starting with a 5-min warm-up, 40-min main training, and end up with a 5-min cool-down. The training details of each group were described as following:

#### 2.3.1 Resistance + aerobic training group (RAeT)

The RAeT included resistance training (RT) and aerobic training (AeT) program. The RT focused on lower extremity workout, including leg press, hamstring curls, and calf raises (Kluding et al., 2011). The participants started with intensity at 12 RM and finished with intensity at 8 RM after 3 weeks. Each action consisted of 8 repetitions, with 2 sets.

The AeT was composed by cycling on stationary bikes in the first week, stepping on steppers in 2nd week, and running on treadmills in the last week. Throughout each session, participants wore portable heart rate monitors, allowing the examiner to monitor intensity and adjust workload in real-time. The target intensity was set at 60–75% of the heart rate reserve (HRR), an intensity range previously shown to benefit cognitive function (Chen et al., 2014). The Karvonen formula was used for calculation: HRTarget = (HRmax – HRrest) * (60–75%) ± HRrest. Individual HRmax was predicted using the formula 220 – age. Each AeT session lasted 25 min, with brief rest periods permitted as needed to maintain the target intensity throughout the session.

#### 2.3.2 Agility training group (AT)

The AT protocol included technical drills, pattern running, and reactive agility exercises (Supplementary Table 1 and Supplementary Figures 1–5). The training protocol progressed with intensity, complexity or velocity every week (Supplementary Table 2). During training, the instructor emphasized the smoothness of motions first, then the speed of motions, if the participants were more proficient in these training tasks.

### 2.4 Outcome measures

#### 2.4.1 Executive function

The EF was assessed using PsyToolKit, an online software for psychological experiments (Stoet, 2017; Stoet, 2010). The assessment included:

1. N-back task (3-back): This task assessed working memory (Jacola et al., 2014). Participants viewed a sequence of alphabets and had 3 s to respond if the current letter matched the one from 3 trials prior. Correct response time and accuracy were calculated.
2. Stroop test: This 40-trial test evaluated selective attention and conflict resolution (Diamond, 2013). Participants were required to name the ink color of a presented word. Trials were either “congruent” (e.g., the word “RED” in red ink) or “incongruent” (e.g., the word “YELLOW” in green ink), with a 3-s response limit per trial. Response times and accuracies for both conditions were calculated.
3. Wisconsin card sort test (WCST): This 60-trial test was used to assess cognitive flexibility, specifically mental set-shifting (Kato et al., 2018). Participants sorted cards according to a rule (color, shape, or number) that changed every 10 trials without notice. The error rate, perseverative error rate, and conceptual-level response rate were calculated based on their performance.

#### 2.4.2 Single and dual task walking performance

Dual task walking is a common method to assess the motor component of EF (Yogev-Seligmann et al., 2008). Participants were instructed to subtract by 7 serially from a 3-digit number while walking at their fastest speed in dual task walking condition (Beauchet et al., 2005). In single walking condition, participants were asked to walk at their fastest speed and just focus on the walking task. For both conditions, spatial–temporal parameters (walking speed, cadence, and stride length) were recorded using three inertial sensors (Opal, APDM Inc.) and analyzed with Mobility Lab™ software. During DT walking, the speed and accuracy of the arithmetic task were also recorded. Each walking condition repeated two times, respectively, with a random order.

#### 2.4.3 Brain activity

**Figure 2:**
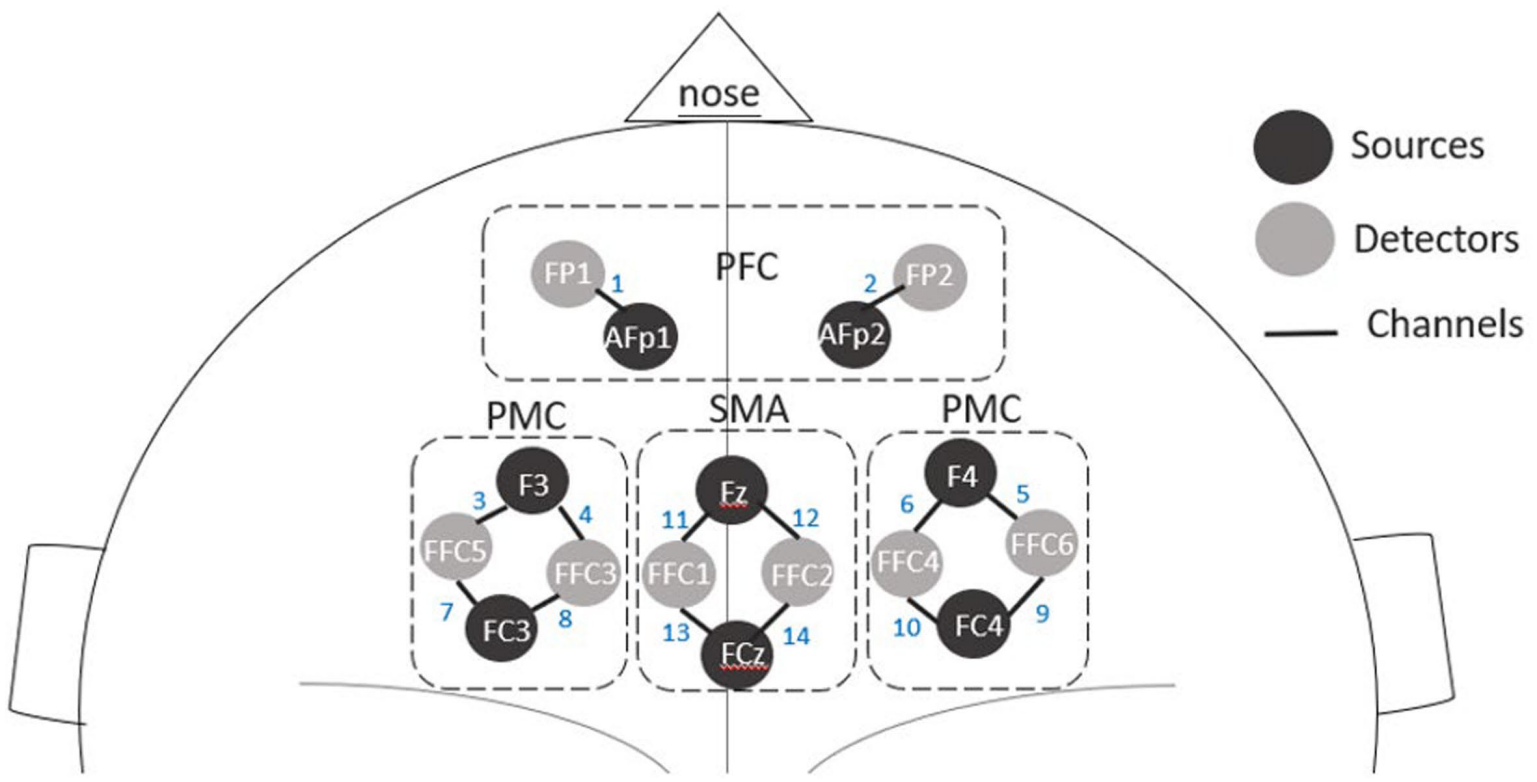
Configuration of fNIRS optodes. PFC, prefrontal cortex; PMC, premotor cortex; SMA, supplementary motor area.

Cortical activation was monitored during each EF tests and walking tasks. A multichannel wearable fNIRS imaging system (NIRSport2, NIRx Medical Technologies LLC, Glen Head, NY, United States) was used to detect the hemodynamics of the bilateral prefrontal cortex (PFC), supplementary motor area (SMA), and premotor cortex (PMC). PFC has been shown to play an important role in cognitive function especially EF (Moriguchi and Hiraki, 2013); SMA is responsible for planning motor action and guiding self-initiated motor control (Leff et al., 2011); PMC is an area which integrates information about the target and the body-part to plan a forthcoming action (Hoshi and Tanji, 2000). The instrument exports and receives the near infrared signals in the dual-wavelength (760 and 850 nm) by 8 LED light sources and 8 detectors, which were attached on subjects’ heads (Figure 2). The fNIRS head cap is designed to be compatible with the international 10–5 system, which defines standard surface positions for a human head with approximately 3.0 cm between any 2 adjacent positions (Cassilhas et al., 2012).

The fNIRS data processing was performed with the HOMER2 package and followed recent recommendations (Liu et al., 2018; Vitorio et al., 2017). The steps included: (a) visual inspection of raw data; (b) rejection of channels with a coefficient of variation (CV) > 15% or trials with a CV > 10%; (c) conversion to optical density via the modified Beer–Lambert law; (d) marking and rejection of motion artifacts; (e) wavelet filtering to remove remaining motion artifacts; (f) bandpass filtering (0.005–0.03 Hz) to remove physiological noise; (g) conversion to concentration changes of oxygenated (HbO) and deoxygenated hemoglobin (HbR); and (h) block averaging of the signal for each task. Brain activation was documented using HbO, as it offers higher sensitivity to cerebral blood flow changes, better detection of task-evoked changes, stronger correlation with the fMRI BOLD response, and is a common practice in recent fNIRS studies (Kinder et al., 2022; Tsou et al., 2025).

### 2.5 Safety and adverse events monitoring

Safety was monitored throughout the intervention. Harms were defined as any adverse events related to the training protocols, including musculoskeletal injuries, falls, or cardiovascular discomfort. The examiner assessed potential harms through real-time observation during sessions and by interviewing participants regarding their physical status at the beginning and end of each visit. No study-related adverse events were reported or observed during the trial.

### 2.6 Statistical analysis

Data were analyzed using SPSS version 25.0 for macOS (SPSS Inc., Chicago, IL, United States). Descriptive statistics are presented as means and standard deviations for parametric data, and medians and interquartile ranges (Q1–Q3) for non-parametric data. As the data were not all normally distributed, non-parametric tests were employed. The Mann–Whitney *U* test was used for between-group comparisons. For within-group comparisons across the three time points (pre-test, post-test, and follow-up), the Friedman test was first applied. If a significant main effect was found, pairwise comparisons were then conducted using the Wilcoxon signed-rank test as a post-hoc analysis. If a significant between-group difference was observed at pre-test, subsequent analyses for that variable were performed on change scores (post-pre and follow-up-pre). Effect sizes for the Mann–Whitney *U* were expressed as *r* (|*r* = *Z* /√*N*|) and interpreted as small (0.1), medium (0.3), or large (0.5). For the Friedman tests, Kendall’s *W* was used, with values categorized as weak (*W* ≤ 0.3), moderate (0.3 ≤ *W* ≤ 0.6), and strong (*W* ≥ 0.6). Missing data were handled by ITT analysis. Significance was set at *p* < 0.05.

## 3 Results

A total of 26 individuals were screened for eligibility. All screened participants met the predefined inclusion criteria and were subsequently enrolled and randomized into the study groups. The participants randomly assigned to the Agility Training (AT) group (*n* = 13) or the Resistance + Aerobic Training (RAeT) group (*n* = 13), as illustrated in Figure 1. At baseline, the two groups were well-matched, with no significant differences in mean age (AT: 22.84 ± 0.98 years; RAeT: 22.84 ± 1.28 years; *p* = 0.093), gender distribution (AT: 5 males/8 females; RAeT: 6 males/7 females; *p* = 0.697), or BMI (AT: 21.18 ± 1.67; RAeT: 20.78 ± 1.98; *p* = 0.696).

### 3.1 Executive function performance

The effects of training on EF are summarized in Tables 1, 2. The AT group demonstrated significant improvements in response time. Specifically, their correct response time on the N-back test decreased from pre-test to the one-month follow-up (*p* = 0.005, *W* = 0.74). Similarly, their response time for incongruent trials on the Stroop test significantly decreased from pre-test to post-test (*p* = 0.016, *W* = 0.65) and to follow-up (*p* = 0.002, *W* = 0.78). A significant between-group difference was noted for Stroop task inhibition at pre-test (*p* = 0.035, *r* = 0.41); however, an analysis of the change scores revealed no significant between-group differences in improvement over time. Regarding accuracy, no statistically significant within-group or between-group differences were found for any EF task (Table 2).

**Table 1:** The results of response time (ms) in the executive function tasks.

	AT (*n* = 13)	RAeT (*n* = 13)	*p*-value (z score)
	Med (Q1–Q3)	Med (Q1–Q3)	
N back-Correct response time			
Pre-test	968.61 (894.87–1081.13)	1033.88 (801.89–1224.67)	0.724 (−0.385)
Post-test	885.94 (794.16–973.55)	1054.45 (814.88–1183.81)	0.139 (−1.513)
Follow-up	822.21 (713.08–986.57)^†^	890.85 (784.10–1099.78)	0.223 (−1.256)
	Pre-Post *p* = 0.052|Post-F/u *p* = 0.424|**Pre-F/u *p* = 0.005**	*p* = 0.436	
Stroop task-congruent response time			
Pre-test	915.45 (729.11–1046.54)	852.45 (755.19–928.21)	0.684 (−0.454)
Post-test	841.22 (670.39–1030.32)	806.88 (649.81–914.90)	0.481 (−0.756)
Follow-up	721.55 (670.22–902.60)	673.97 (606.43–883.89)	0.393 (−0.907)
	*p* = 0.249	*p* = 0.051	
Stroop task-incongruent response time			
Pre-test	1085.20 (817.84–1143.00)	881.58 (808.43–971.38)	0.116 (−1.590)
Post-test	907.96 (782.87–989.25)^§^	843.70 (741.77–910.24)	0.311 (−1.051)
Follow-up	843.00 (697.15–1010.93)^†^	825.43 (675.21–880.62)	0.448 (−0.795)
	**Pre-Post *p* = 0.016**|Post-F/u p = 0.052|**Pre-F/u *p* = 0.002**	*p* = 0.135	
Stroop task-inhibition control			
Pre-test	157.96 (4.70–239.16)*	10.18 (−52.94–68.06)*	**0.035** (−2.103)
Post-test	40.16 (−0.78–119.25)	29.63 (−43.48–71.85)	0.362 (−0.946)
Follow-up	84.11 (−52.94–68.06)	69.59 (33.25–426.91)	0.960 (−0.077)
	*p* = 0.434	*p* = 0.126	
Stroop task-inhibition control change value			
Post-pre	−103.01 (−173.95–26.01)	0 (−79.08–99.36)	0.270 (−1.128)
F/u-pre	−51.30 (−174.09–23.16)	44.17 (−123.09–149.21)	0.208 (−1.282)
	*p* = 1.000	*p* = 1.000	

†, significant within-group difference from pretest to follow-up; §, significant within-group difference from pretest to post-test; ‡, significant within-group difference from post-test to follow-up; *, significant between-group difference.

F/u, follow-up; RAeT, resistance + aerobic training; AT, agility training. Bold values indicate statistically significant results (*p* < 0.05).

**Table 2:** The results of accuracy in the executive function tasks.

	AT (*n* = 13)	RAeT (*n* = 13)	*p*-value (*z* score)
	Med (Q1–Q3)	Med (Q1–Q3)	
N back-Correct response rate (%)			
Pre-test	80.00 (62.50–90.00)	80.00 (75.00–87.50)	0.516 (−0.673)
Post-test	80.00 (70.00–85.00)	80.00 (75.00–92.50)	0.533 (−0.649)
Follow-up	85.00 (72.50–95.00)	85.00 (77.50–97.50)	0.261 (−1.147)
	*p* = 0.771	*p* = 0.727	
Stroop task-correct response rate of congruent (%)			
Pre-test	100.00 (95.45–100.00)	100.00 (100.00–100.00)	0.797 (−0.485)
Post-test	10.00 (90.00–100.00)	100.00 (100.00–100.00)	0.104 (−1.694)
Follow-up	100.00 (95.83–100.00)	100.00 (95.45–100.00)	0.858 (−0.104)
	*p* = 0.317	*p* = 0.673	
Stroop task-correct response rate of incongruent (%)			
Pre-test	96.55 (91.26–100.00)	96.67 (94.88–100.00)	0.445 (−0.787)
Post-test	96.77 (92.39–100.00)	100.00 (96.21–100.00)	0.424 (−0.822)
Follow-up	93.94 (91.38–100.00)	96.77 (96.49–100.00)	0.367 (−0.925)
	*p* = 0.879	*p* = 0.954	
Wisconsin card sort test-error rate (%)			
Pre-test	15.00 (10.00–17.50)	13.33 (12.91–15.41)	0.768 (−0.312)
Post-test	13.33 (12.50–17.50)	13.33 (10.00–17.50)	0.464 (−0.755)
Follow-up	13.33 (10.83–16.66)	13.33 (10.83–15.00)	0.789 (−0.288)
	*p* = 0.890	*p* = 0.993	
Wisconsin card sort test-perseverative error rate (%)			
Pre-test	10.00 (8.33–10.00)	10.00 (8.33–11.66)	0.603 (−0.567)
Post-test	10.00 (8.33–11.66)	10.00 (8.33–11.66)	0.817 (−0.240)
Follow-up	10.00 (8.33–10.00)	10.00 (9.16–10.83)	0.347 (−1.016)
	*p* = 0.791	*p* = 0.954	
Wisconsin card sort test-conceptual-level response rate (%)			
Pre-test	83.33 (78.33–90.00)	85.00 (80.00–87.50)	0.759 (−0.338)
Post-test	85.00 (80.83–87.50)	85.00 (79.16–89.16)	0.730 (−0.362)
Follow-up	85.00 (80.83–89.16)	86.66 (82.50–87.50)	0.600 (−0.545)
	*p* = 0.699	*p* = 1.000	

†, significant within-group difference from pretest to follow-up; §, significant within-group difference from pretest to post-test; ‡, significant within-group difference from post-test to follow-up; *, significant between-group difference.

RAeT, resistance + aerobic training; AT, agility training.

### 3.2 Single and dual task walking performance

In the single-task walking condition, the RAeT group exhibited a significantly longer stride length than the AT group at all three time points (*p* ≤ 0.021, *r* = 0.45–0.54), though the change in stride length over time did not differ significantly between groups (Table 3).

**Table 3:** The results of gait performance in the single-task (ST) and dual-task (DT) walking conditions.

	AT (*n* = 13)	RAeT (*n* = 13)	*p*-value (*z* score)
	Med (Q1–Q3)	Med (Q1–Q3)	
ST-Speed (m/s)			
Pre-test	1.65 (1.56–1.75)	1.71 (1.63–1.81)	0.223 (−1.256)
Post-test	1.66 (1.57–1.75)	1.73 (1.64–1.88)	0.208 (−1.284)
Follow-up	1.68 (1. 59–1.77)	1.75 (1.66–1.85)	0.128 (−1.539)
	*p* = 0.897	*p* = 0.383	
ST-Cadence (step/min)			
Pre-test	133.29 (130.39–145.13)	132.58 (122.36–141.72)	0.362 (−0.949)
Post-test	136.94 (132.34–145.58)	133.77 (121.91–143.46)	0.448 (−0.795)
Follow-up	135.66 (130.44–145.97)	133.21 (124.86–139.30)	0.390 (−0.897)
	*p* = 0.798	*p* = 0.751	
ST-Stride length (m)			
Pre-test	1.44 (1.37–1.55)*	1.57 (1.49–1.67)*	**0.008 (−2.591)**
Post-test	1.43 (1.41–1.55)*	1.56 (1.52–1.66)*	**0.021 (−2.284)**
Follow-up	1.45 (1.38–1.52)*	1.56 (1.50–1.64)*	**0.005 (−2.744)**
	*p* = 0.421	*p* = 0.652	
ST-Stride length change value (m)			
Post-Pre	0.040 (−0.006–0.072)	−0.002 (−0.031–0.025)	0.181 (−1.360)
F/u-Pre	0.000 (−0.027–0.052)	0.002 (−0.036–0.030)	1.000 (0.000)
	*p* = 0.349	*p* = 0.914	
DT-Speed (m/s)			
Pre-test	1.55 (1.44–1.63)	1.70 (1.55–1.76)	0.122 (−1.564)
Post-test	1.62 (1.44–1.67)	1.70 (1.57–1.74)	0.099 (−1.667)
Follow-up	1.57 (1.52–1.73)	1.72 (1.63–1.80)	0.098 (−1.668)
	*p* = 0.123	*p* = 0.084	
DT-Cadence (step/min)			
Pre-test	129.98 (126.63–136.32)	129.26 (116.16–137.79)	0.579 (−0.590)
Post-test	131.40 (129.58–138.07)	129.65 (119.21–142.25)	0.390 (−0.897)
Follow-up	130.93 (128.75–142.98)	133.16 (120.40–134.68)	0.448 (−0.795)
	Pre-Post *p* = 0.052|Post-F/u *p* = 0.519|**Pre-F/u p = 0.024**	*p* = 0.383	
DT-Stride length (m)			
Pre-test	1.37 (1.31–1.43)*	1.49 (1.46–1.63)*	**0.000 (−3.360)**
Post-test	1.41 (1.33–1.44)*	1.56 (1.50–1.63)*	**0.001 (−3.154)**
Follow-up	1.40 (1.36–1.47)*	1.56 (1.48–1.65)*	**0.002 (−2.950)**
	*p* = 0.127	p = 0.727	
DT-Response speed (/s)			
Pre-test	0.24 (0.20–0.27)	0.26 (0.23–0.30)	0.179 (−1.365)
Post-test	0.25 (0.23–0.28)	0.29 (0.25–0.31)^§^	0.082 (−1.753)
Follow-up	0.24 (0.21–0.29)*	0.28 (0.25–0.32)^*†^	**0.045 (−2.004)**
	*p* = 0.061	**Pre-Post *p* = 0.018**|Post-F/u *p* = 0.576|**Pre-F/u *p* = 0.008**	
DT-Correct response ratio (%)			
Pre-test	93.33 (88.38–98.07)	96.87 (93.33–100.00)	0.217 (−1.269)
Post-test	94.11 (91.17–98.52)	97.05 (94.34–100.00)	0.118 (−1.581)
Follow-up	95.23 (90.09–100.00)	97.22 (95.43–100.00)	0.094 (−1.703)
	*p* = 0.586	*p* = 0.056	
Dual task cost (%)			
Pre-test	−7.71 (−12.38– −4.13)	−2.61 (−7.36– −1.10)	0.315 (−1.058)
Post-test	−4.91 (−13.27– −1.17)	−4.08 (−7.80– −1.77)	0.579 (−0.605)
Follow-up	−3.61 (−8.64– −1.58)	−2.36 (−4.75– −0.62)	0.315 (−1.058)
	*p* = 0.287	*p* = 0.123	
DT-Stride length change value (m)			
Post-pre	0.057 (0.005–0.080)	0.002 (−0.051–0.083)	0.397 (−0.872)
F/u-pre	0.020 (−0.027–0.122)	0.000 (−0.041–0.125)	0.772 (−0.308)
	*p* = 0.925	*p* = 0.332	

†, significant within-group difference from pretest to follow-up; §, significant within-group difference from pretest to post-test; ‡, significant within-group difference from post-test to follow-up; *, significant between-group difference.

ST, single task walking; DT, dual task walking; F/u, follow-up; RAeT, resistance + aerobic training; AT, agility training. Bold values indicate statistically significant results (*p* < 0.05).

Performance during the dual-task walking condition revealed several effects (Table 3). For gait parameters, the AT group showed a significant improvement in cadence from pre-test to follow-up (*p* = 0.024, *W* = 0.62). The RAeT group again maintained a longer stride length across all assessments (*p* ≤ 0.002, *r* = 0.58–0.66), but without a significant difference in the change over time. Regarding the cognitive component of the task, the RAeT group’s response speed became significantly faster from pre-test to post-test (*p* = 0.018, *W* = 0.65) and to follow-up (*p* = 0.008, *W* = 0.72). Consequently, at the follow-up assessment, the RAeT group’s response speed was significantly faster than that of the AT group (*p* = 0.045, *r* = 0.39).

### 3.3 Brain activation while EF tests and walking conditions

**Figure 3:**
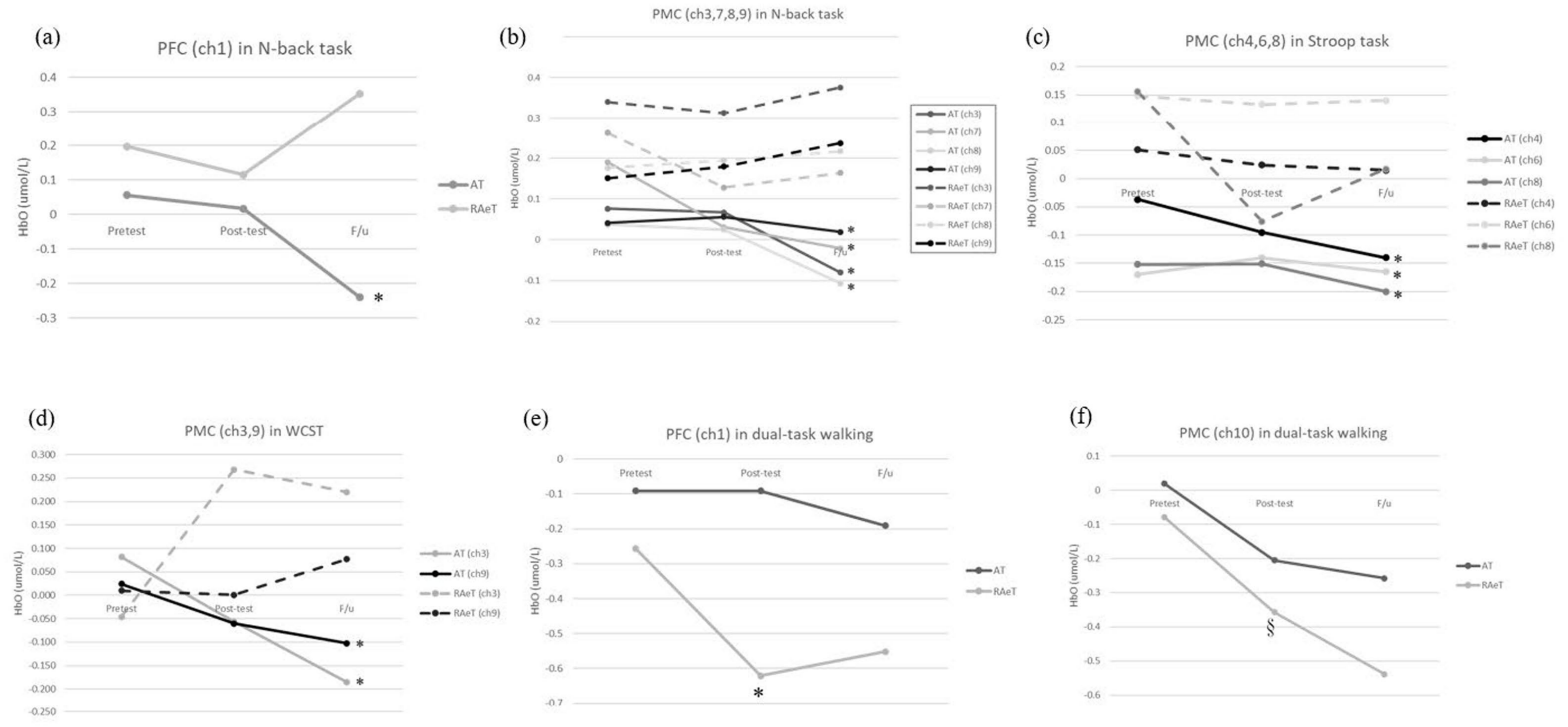
Brain activation of **(a)** PFC (channel 1) during N-back task; **(b)** PMC (channel 3, 7–9) during N-back task; **(c)** PMC (channel 4, 6, 8) during Stroop task; **(d)** PMC (channel 3, 9) during WCST; **(e)** PFC (channel 1) during dual-task walking; **(f)** Brain activation of PMC (channel 10) during dual-task walking. *Significant between-group difference. F/u, follow-up; RAeT, resistance + aerobic training.

Brain activation patterns, indicated by HbO changes, differed between the groups during various tasks (Supplementary Tables 3–5). During EF tasks, the AT group generally showed lower brain activation than the RAeT group, particularly at follow-up. This was evident in the N-back task, where the AT group showed significantly lower activation in the PFC and PMC (*p* ≤ 0.035, *r* ≥ 0.42, Figures 3a,b); the Stroop task, with lower AT group activation in the PMC (*p* ≤ 0.040, *r* ≥ 0.41, Figure 3c); and the WCST, again with lower AT group activation in the PMC at post-test and follow-up (*p* ≤ 0.014, *r* ≥ 0.49, Figure 3d).

In contrast, during dual-task walking, the RAeT group showed instances of lower activation. Specifically, the RAeT group had lower PFC activation than the AT group at follow-up (*p* = 0.046, *r* = 0.40, Figure 3e) and also demonstrated a significant decrease in its PMC activation from pre-test to post-test (*p* = 0.016, *W* = 0.47, Figure 3f). Although most channel activations did not reach statistical significance, a general trend of decreasing brain activation from pre-test to post-test was observed in both groups, which trended back toward baseline levels at the one-month follow-up (Supplementary Tables 3–7).

## 4 Discussion

To our knowledge, this is the first study to investigated and compare the effect of agility training (AT) and combined resistance-aerobic training (RAeT) on EF, dual task walking and brain activation in healthy adults. Our preliminary findings suggest distinct cognitive-motor profiles associated with each training type. Specifically, AT demonstrated particular strengths in enhancing reactive, unpredictable cognitive control, as evidenced by improved inhibitory control and working memory performance; while RAeT preferentially improved stable, rhythmic cognitive-motor integration tasks, reflected in enhanced dual-task cognitive response speed. These behavioral differences were accompanied by task-specific patterns of neural adaptation.

The observed benefit of agility training on working memory and inhibitory control are consistent with previous research on cognitive function enhancement through sport training or open skill exercises (Srinivas et al., 2021; Ferreira et al., 2022). Agility training, by its nature, is a multi-faceted process. Drills such as pattern running require participants to hold, execute, and continuously update sequences of movements, directly engaging the core components of working memory. Simultaneously, its reactive components demand that individuals constantly perceive, react to, and inhibit inappropriate responses to external stimuli (Ferreira et al., 2022; Di Russo et al., 2010), such as in the reactive drills used in our protocol. This sustained demand on selective attention and response inhibition likely underlies the significant improvements observed in the AT group’s Stroop task performance, which is consistent with findings from studies of athletes in open-skill sports (Wang et al., 2013). Importantly, these behavioral improvements were accompanied by lower brain activation in the prefrontal and premotor cortices during EF tasks compared to the RAeT group. This phenomenon, characterized by reduced cortical recruitment, suggests a potential optimization of cognitive control networks. This pattern may reflect a more economical neural response, where the brain executes tasks with fewer neural resources after training, a phenomenon often observed in highly trained athletes who exhibit more focused activation patterns (Guo et al., 2017; Causse et al., 2017).

In contrast, the primary behavioral improvement in the RAeT group was a significantly faster cognitive response speed during dual-task walking. This is well-supported by previous literature indicating that both resistance and aerobic training can enhance information processing and reaction times (Herold et al., 2019; Chang et al., 2017; Coetsee and Terblanche, 2017). Importantly, the neural adaptations were task-dependent. While the AT group showed greater reduced cortical recruitment during sedentary EF tasks, the pattern reversed during dual-task walking. In the walking condition, the RAeT group exhibited not only faster cognitive performance but also lower activation in specific PFC and PMC channels. This finding suggests that RAeT may induce a more streamlined neural response specific to integrated cognitive-motor tasks (Guo et al., 2017; Coetsee and Terblanche, 2017). We speculate that RAeT enhances the automaticity of walking, reducing the need for conscious supervision by the prefrontal cortex. As walking becomes more automatic, cognitive resources can be reallocated to the secondary arithmetic task, resulting in faster performance. This highlights that the specificity of exercise-induced neural adaptations: agility training appears to optimize top-down, reactive cognitive control, whereas RAeT may be more effective at reducing the neural demand of stable, integrated cognitive-motor behaviors.

In addition to the specific adaptations that distinguished the two training groups, it is noteworthy that a general, though non-significant, trend was observed. Both groups demonstrated a tendency toward decreased brain activation immediately post-intervention, which trended back toward baseline at the one-month follow-up. This may suggest that any structured exercise imparts a temporary, non-specific reduction in cortical activation. The “wash-out” effect at follow-up underscores that the benefits of exercise training likely require continued practice to be maintained, a finding consistent with long-term study (Talamonti et al., 2021). These results highlight the importance of continued practice to maintain the neurofunctional benefits derived from exercise training.

### 4.1 Limitations

Although this study provides insights into the effects of agility training and combined resistance-aerobic training on EF, dual task walking, and brain plasticity in healthy adults, several limitations should be noted. First, the relatively small sample size may limit the statistical power to detect meaningful effects and compromises the generalizability of our findings. Despite this limitation, post-hoc analysis revealed large effect sizes (r values ranging from 0.4 to 0.7 and *W* higher than 0.6) for the primary outcomes. This suggests that the intervention-induced changes were robust and that the study was sufficiently powered to detect meaningful differences, notwithstanding its pilot nature. Future research with larger cohorts is warranted to confirm these results. Future research with larger cohorts is needed to confirm these results. Second, although participants were instructed to maintain their habitual lifestyle patterns, their routine physical activity levels outside the laboratory were not strictly monitored during the 3-week intervention. Although we recruited individuals with low baseline activity to enhance homogeneity, these uncontrolled lifestyle factors may have introduced variance in prefrontal activation and executive function. Future studies should utilize objective monitoring tools to better isolate the intervention’s effects. Third, a ceiling effect was observed for cognitive flexibility as measured by the WCST. The WCST was originally developed to assess patients with neuropsychiatric conditions, and may lack sensitivity for detecting changes in high-functioning young adults. Indeed, our participants’ baseline scores frequently approached the maximum possible values, exceeding published age norm (Shan et al., 2008), which may have masked potential improvements in cognitive flexibility. Future studies should incorporate more demanding cognitive flexibility tasks, such as the Task-Switching Paradigm or the Comprehensive Trail Making Test, to better capture variability in high-functioning cohorts. Fourth, though we observed reduced HbO activation, it may also reflect decreased activation due to increased familiarity with the testing tasks in pre-test, post-test and follow-up test. Future studies may need to incorporate analyses of behavior–brain correlation analyses to further validate the functional significance of these neural changes. Fifth, our analyses focused on HbO signals, as HbO typically exhibits a higher signal-to-noise ratio and greater sensitivity to task-related cortical activation compared to HbR in fNIRS measurements (Kinder et al., 2022). While consistent with existing literature, this approach may not fully capture neurovascular coupling. Additionally, the inherently lower spatial resolution of fNIRS compared to fMRI limits the precise localization of cortical activation. Thus, our findings regarding anatomical precision should be interpreted with caution. Sixth, the absence of muscle strength measurements limits our ability to distinguish whether dual-task improvements stemmed from peripheral muscular adaptations or central cognitive enhancement. While agility training can increase strength (Lichtenstein et al., 2020), such physical gains may reduce the cognitive load of motor control, thereby facilitating motor automaticity and resource reallocation. Future studies should incorporate objective assessments, such as isokinetic dynamometry, to clarify the specific contributions of muscular versus neural adaptations. Finally, the lack of blinding may have introduced potential bias and should be considered when interpreting the results. Collectively, the results of this study provide preliminary evidence of exercise-induced benefits that likely reflect short-term functional adaptations rather than definitive or permanent neurophysiological changes.

## 5 Conclusion

This study provides preliminary evidence a 3-week intervention of either agility training or combined resistance-aerobic training can induce positive short-term adaptation on executive function, dual-task walking, and brain activation in healthy adults. Notably, these two modalities appear to facilitate these improvements through distinct functional pathways. Agility training may enhance cognitive control by promoting a more economical neural recruitment pattern, whereas combined training improves dual-task performance, likely by facilitating motor automaticity.

These initial findings offer practical insights for exercise program development in both clinical and athletic settings. Agility training appears particularly beneficial where rapid cognitive control and flexible responses are required, while resistance-aerobic training may better support tasks involving stable, rhythmic cognitive-motor integration. Given its engaging and equipment-free nature, agility training may also serve as a feasible and cost-effective intervention—especially in resource-limited environments. However, given the small sample size and brief intervention duration, the observed neural changes likely reflect short-term functional adaptations rather than stable neuroplastic restructuring. Future large-scale studies are warranted to confirm and extend these findings, and to further elucidate the underlying neural mechanisms of different training modalities.

## Data Availability

The raw data supporting the conclusions of this article will be made available by the authors, without undue reservation.
