# Supplementary material for: Distinct neural mechanisms underlying the effects of agility and resistance-aerobic training on executive function and gait in young adults
Source: Front Psychol. 2026 Jun 10;17:1743946. doi: 10.3389/fpsyg.2026.1743946 (PMC13290560; doi:10.3389/fpsyg.2026.1743946)
Supplement: Supplementary file 1 [file Table_1.DOCX]

Supplementary Material

# Supplementary Tables

**Supplementary Table 1. Agility training content**

| Training item | Training content |
| --- | --- |
| Technical drills  (Low CI) | 1. Ladder drill (different patterns) 2. Hurdle drill (different patterns) 3. Plyometric Box Drill 4. Shuttle run |
| Pattern running  (Moderate CI) | 1. Cone drill (different patterns, distances or colors) 2. Agility ball drill 3. Change of direction (COD) run 4. Cutting manouvers |
| Reactive agility training  (High CI) | 1. Mirror drill (lateral/sprint/backpedal) 2. Catching-ball drill (in front of/behind/throw to the wall) |

Abbreviations: CI, contextual interference

**Supplementary Table 2. The training protocol in AT group**

|  | CI | Drills | Repetitions | Rest Intervals (seconds) |
| --- | --- | --- | --- | --- |
| Week1 |  |  |  |  |
| Session 1 | Low | 8 | 2-3 | 30 |
| Session 2 |  | 8 | 3-5 | 45 |
| Session 3 |  | 8 | 3-5 | 45 |
| Week2 |  |  |  |  |
| Session 4 |  | 6 | 2-3 | 30 |
| Session 5 | Moderate | 6 | 3-5 | 45 |
| Session 6 |  | 6 | 3-5 | 45 |
| Week 3 |  |  |  |  |
| Session 7 |  | 4 | 2-3 | 30 |
| Session 8 | High | 4 | 3-5 | 45 |
| Session 9 |  | 4 | 3-5 | 45 |

Abbreviations: CI, contextual interference

**Supplementary Table 3. Brain activation indicated by HbO in different channels under N-back task**

| **N-back task** | **AT** | **RAeT** |  |
| --- | --- | --- | --- |
|  | **Med (Q1-Q3)** | **Med (Q1-Q3)** |  |
| PFC (ch1: S1D1) | | |  |
| Pre-test | 0.056 (-0.130-0.241) | 0.197 (-0.247-0.424) | p=0.437 (z=-0/816) |
| Post-test | 0.017 (-0.130-0.170) | 0.115 (-0.348-0.377) | p=0.936 (z=-0.109) |
| **Follow-up** | **-0.240 (-0.474-0.129)*** | **0.353 (0.028-0.570)*** | **p=0.008 (z=-2.611)** |
|  | p= 0.514 | p= 0.434 |  |
| PFC (ch2: S2D2) | | |  |
| Pre-test | 0.152 (-0.042-0.344) | 0.136 (-0.258-0.235) | p=0.769 (z=-0.326) |
| Post-test | 0.143 (-0.046-0.328) | 0.204 (-0.534-0.348) | p=0.810 (z=-0.272) |
| Follow-up | 0.139 (-0.242-0.185) | 0.312 (0-0.472) | p=0.098 (z=-1.686) |
|  | p=0.801 | p=0.654 |  |
| PMC (ch3: S3D3) | | |  |
| Pre-test | 0.076 (-0.401-0.407) | 0.340 (0.041-0.487) | p=0.225 (z=-1.251) |
| Post-test | 0.067 (-0.264-0.168) | 0.313 (-0.214-0.484) | p=0.077 (z=-1.795) |
| **Follow-up** | **-0.080 (-0.385-0.372)*** | **0.376 (0.018-0.651)*** | **p=0.022 (z=-2.284)** |
|  | p= 0.806 | p= 0.249 |  |
| PMC (ch4:S3D5) | | |  |
| Pre-test | 0.146 (-0.495-0.398) | 0.156 (-0.062-0.326) | p=0.689 (z=-0.435) |
| Post-test | 0.018 (-0.270-0.145) | 0.183 (-0.218-0.362) | p=0.110 (z=-1.632) |
| Follow-up | 0.042 (-0.210-0.329) | 0.198 (0.025-0.454) | p=0.152 (z=-1.465) |
|  | p= 0.393 | p= 0.751 |  |
| PMC (ch7: S5D3) | | |  |
| Pre-test | 0.190 (-0.072-0.427) | 0.262 (-0.137-0.459) | p=0.689 (z=-0.435) |
| Post-test | 0.031 (-0.085-0.186) | 0.128 (-0.040-0.458) | p=0.320 (z=-1.033) |
| **Follow-up** | **-0.021(-0.394-0.233)*** | **0.164 (-0.091-0.520)*** | **p=0.026 (z=-2.230)** |
|  | p= 0.353 | p=0.978 |  |
| PMC (ch8: S5D5) | | |  |
| Pre-test | 0.037 (-0.257-0.223) | 0.176 (-0.093-0.493) | p= 0.285 (z=-1.088) |
| Post-test | 0.025 (-0.270-0.127) | 0.194 (-0.185-0.311) | p= 0.168 (z=-1.414) |
| **Follow-up** | **-0.107 (-0.443-0.067)*** | **0.217 (-0.021-0.429)*** | **p= 0.008 (z=-2.611)** |
|  | p= 0.268 | p= 0.856 |  |
| PMC (ch5: S4D4) | | |  |
| Pre-test | 0.062 (-0.199-0.278) | 0.225 (-0.432-0.388) | p= 0.689 (z=-0.435) |
| Post-test | 0.028 (-0.194-0.166) | 0.118 (-0.171-0.399) | p= 0.538 (z=-0.653) |
| Follow-up | -0.047 (-0.517-0.240) | 0.233 (0.039-0.566) | p= 0.068 (z=-1.849) |
|  | p= 0.806 | p= 0.978 |  |

| PMC (ch6: S4D6) | | |  |
| --- | --- | --- | --- |
| Pre-test | 0.032 (-0.275-0.112) | 0.127 (-0.248-0.274) | p= 0.168 (z=-1.414) |
| Post-test | 0.064 (-0.223-0.255) | 0.024 (-0.160-0.185) | p= 0.810 (z=-0.272) |
| Follow-up | -0.098 (-0.164-0.120) | 0.262 (-0.030-0.428) | p= 0.060 (z=-1.904) |
|  | p= 0.549 | p= 0.287 |  |
| PMC (ch9: S6D4) | | |  |
| Pre-test | 0.041 (-0.011-0.149) | 0.151 (-0.179-0.360) | p=0.205 (z=-1.305) |
| Post-test | 0.056 (-0.099-0.129) | 0.179 (-0.168-0.379) | p=0.347 (z=-0.979) |
| **Follow-up** | **0,019 (-0.283-0.188)*** | **0.237 (-0.011-0.369)*** | **p=0.035 (z=-2.121)** |
|  | p=0.549 | p=0.856 |  |
| PMC (ch10: S6D6) | | |  |
| Pre-test | 0.020 (-0.272-0.171) | 0.059 (-0.293-0.357) | p=0.650 (z=-0.490) |
| Post-test | 0.005 (-0.327-0.185) | 0.084 (-0.205-0.349) | p=0.503 (z=-0.707) |
| Follow-up | -0.085 (-0.193-0.165) | 0.216 (-0.073-0.441) | p=0.110 (z=-1.632) |
|  | p=0.806 | p=0.978 |  |
| SMA (ch11: S7D7) | | |  |
| Pre-test | -0.078 (-0.247-0.126) | 0.088 (-0.066-0.209) | p=0.225 (z=-1.251) |
| Post-test | -0.014 (-0.247-0.057) | 0.193 (-0.204-0.325) | p=0.152 (z=-1.469) |
| Follow-up | -0.158 (-0.276-0.225) | 0.249 (-0.205-0.419) | p=0.147 (z=-1.197) |
|  | p=0.924 | p=0.500 |  |
| SMA (ch13: S8D7) | | |  |
| Pre-test | -0.101 (-0.290-0.355) | 0.128 (-0.195-0.576) | p=0.205 (z=-1.305) |
| Post-test | -0.090 (-0.238-0.079) | 0.091 (-0.260-0.353) | p=0.186 (z=-1.360) |
| Follow-up | -0.198 (-0.433-0.050) | 0.124 (-0.118-0.320) | p=0.205 (z=-1.305) |
|  | p=0.897 | p=0.687 |  |
| SMA (ch12: S7D8) | | |  |
| Pre-test | -0.165(-0.432-0.149) | 0.163 (-0.224-0.293) | p=0.295 (z=-1.088) |
| Post-test | -0.051(-0.432-0.093) | 0.096 (-0.472-0.253) | p=0.437 (z=-0.816) |
| Follow-up | -0.071(-0.230-0.176) | 0.280 (-0.226-0.477) | p=0.137 (z=-1.523) |
|  | p=0.682 | p=0.383 |  |
| SMA (ch14: S8D8) | | |  |
| Pre-test | -0.483 (-0.341-0.139) | -0.108 (-0.193-0.228) | p=0.406 (z=-0.870) |
| Post-test | 0.071 (-0.229-0.146) | -0.108 (-0.198-0.123) | p=0.503 (z=-0.707) |
| Follow-up | -0.158 (-0.412-0.225) | 0.124 (-0.118-0.320) | p=0.270 (z=-1.142) |
|  | p= 0.780 | p=0.500 |  |

†, significant within-group difference from pretest to follow-up; §, significant within-group difference from pretest to post-test; ‡, significant within-group difference from post-test to follow-up; *, significant between-group difference

Abbreviations: RAeT, resistance+aerobic training; AT, agility training; PFC, prefrontal cortex; PMC, premotor cortex; SMA, supplementary motor area

**Supplementary Table 4. Brain activation indicated by HbO in different channels under Stroop task**

| **Stroop task** | **AT** | **RAeT** |  |
| --- | --- | --- | --- |
|  | **Med (Q1-Q3)** | **Med (Q1-Q3)** |  |
| PFC (ch1: S1D1) | | |  |
| Pre-test | 0.016 (-0.253-0.271) | -0.092 (-0.316-0.274) | p=0.990 (z=-0.027) |
| Post-test | -0.035 (-0.310-0.288) | 0.033 (-0.251-0.212) | p=0.769 (z=-0.326) |
| Follow-up | -0.153 (-0.380-0.151) | 0.050 (-0.386-0.326) | p=0.470 (z=-0.761) |
|  | p= 0.676 | p=0.978 |  |
| PFC (ch2: S2D2) | | |  |
| Pre-test | -0.010 (-0.161-0.446) | 0.144 (-0.304-0.440) | p=0.936 (z=-0.109) |
| Post-test | -0.006 (-0.425-0.277) | 0.180 (-0.228-0.371) | p=0.470 (z=-0.761) |
| Follow-up | -0.146 (-0.326-0.181) | 0.154 (-0.246-0.391) | p=0.406 (z=-0.870) |
|  | p=0.950 | p=0.978 |  |
| PMC (ch3: S3D3) | | |  |
| Pre-test | -0.085 (-0.333-0.152) | 0.418 (-0.156-0.598) | p=0.168 |
| Post-test | -0.115 (-0.183-0.034) | 0.145 (-0.276-0.301) | p=0.205 |
| Follow-up | -0.219 (-0.325-0.219) | 0.106 (-0.172-0.466) | p=0.098 |
|  | p=0.947 | p=0.856 |  |
| PMC (ch4:S3D5) | | |  |
| Pre-test | -0.037 (-0.477-0.340) | 0.051 (-0.367-0.533) | p=0.769 (z=-0.326) |
| Post-test | -0.095 (-0.209-0.096) | 0.024 (-0.239-0.106) | p=0.810 (z=-0.272) |
| **Follow-up** | **-0.140 (-0.307- -0.012)*** | **0.015 (-0.071-0.305)*** | **p=0.040 (z=-2.067)** |
|  | p= 0.491 | p=0.654 |  |
| PMC (ch7: S5D3) | | |  |
| Pre-test | 0.003 (-0.155-0.232) | 0.260 (-0.199-0.393) | p= 0.574 (z=-0.598) |
| Post-test | -0.032 (-0.162-0.129) | 0.006 (-0.150-0.075) | p= 0.979 (z=-0.054) |
| Follow-up | -0.108 (-0.313-0.002) | 0.026 (-0.089-0.386) | p= 0.060 (z=-1.904) |
|  | p= 0.763 | p= 0.249 |  |
| PMC (ch8: S5D5) | | |  |
| Pre-test | -0.152 (-0.218-0.190) | 0.156 (-0.353-0.379) | p= 0.538 (z=-0.653) |
| Post-test | -0.151 (-0.393-0.126) | -0.076 (-0.242-0.243) | p= 0.503 (z=-0.707) |
| **Follow-up** | **-0.200 (-0.398-0.137)*** | **0.017 (-0.145-0.337)*** | **p= 0.005 (z=-2.774)** |
|  | p= 0.112 | p= 0.500 |  |
| PMC (ch5: S4D4) | | |  |
| Pre-test | -0.130 (-0.196-0.305) | 0.182 (-0.657-0.409) | p=0.894 (z=-0.163) |
| Post-test | -0.238 (-0.455-0.090) | 0.171 (-0.202-0.318) | p=0.123 (z=-1.577) |
| Follow-up | -0.257 (-0.389-0.025) | 0.081 (-0.332-0.183) | p=0.098 (z=-1.686) |
|  | p= 0.353 | p= 0.856 |  |
| PMC (ch6: S4D6) | | |  |
| Pre-test | -0.170 (-0.256-0.259) | 0.148 (-0.116-0.394) | p= 0.346 (z=-0.926) |
| Post-test | -0.140 (-0.297-0.222) | 0.133 (-0.095-0.246) | p= 0.347 (z=-0.979) |
| **Follow-up** | **-0.165 (-0.286-0.005)*** | **0.140 (-0.087-0.290)*** | **p= 0.019 (z=-2.339)** |
|  | p= 0.520 | p= 0.856 |  |

| PMC (ch9: S6D4) | | |  |
| --- | --- | --- | --- |
| Pre-test | -0.076 (-0.293-0.088) | 0.053 (-0.160-0.404) | p= 0.186 (z=-1.360) |
| Post-test | -0.078 (-0.181-0.074) | 0.043 (-0.186-0.227) | p= 0.347 (z=-0.979) |
| Follow-up | -0.063 (-0.302-0.079) | 0.020 (-0.047-0.150) | p= 0.247 (z=-1.197) |
|  | p= 0.806 | p= 0.978 |  |
| PMC (ch10: S6D6) | | |  |
| Pre-test | -0.019 (-0.276-0.286) | 0.085 (-0.444-0.483) | p=0.538 (z=-0.653) |
| Post-test | -0.169 (-0.272-0.148) | 0.089 (-0.151-0.253) | p=0.320 (z=-1.033) |
| Follow-up | -0.124(-0.234-0.021) | 0.013 (-0.110-0.152) | p=0.098 (z=-1.686) |
|  | p=0.682 | p=0.856 |  |
| SMA (ch11: S7D7) | | |  |
| Pre-test | -0.064 (-0.139-0.133) | 0.016 (-0.194-0.316) | p=0.538 (z=-0.653) |
| Post-test | 0.046 (-0.110-0.148) | 0.067 (0.001-0.174) | p=0.503 (z=-0.707) |
| Follow-up | -0.028 (-0.113-0.058) | 0.075 (-0.155-0.207) | p=0.503 (z=-0.707) |
|  | p=0.806 | p=0.856 |  |
| SMA (ch13: S8D7) | | |  |
| Pre-test | -0.088 (-0.385-0.063) | -0.039 (-0.261-0.144) | p=0.810 (z=-0.272) |
| Post-test | -0.024 (-0.176-0.100) | -0.024 (-0.191-0.142) | p=0.728 (z=-0.381) |
| Follow-up | -0,055 (-0.271-0.265) | -0.007 (-0.338-0.079) | p=0.611 (z=-0.544) |
|  | p=0.682 | p=0.856 |  |
| SMA (ch12: S7D8) | | |  |
| Pre-test | -0.046 (-0.257-0.309) | -0.016 (-0.297-0.414) | p=0.894 (z=-0.163) |
| Post-test | 0.043 (-0.318-0.144) | 0.067 (-0.101-0.236) | p=0.650 (z=-0.490) |
| Follow-up | -0.100 (-0.191- -0.033) | 0.150 (-0.139-0.295) | p=0.087 (z=-1.741) |
|  | p=0.319 | p=0.653 |  |
| SMA (ch14: S8D8) | | |  |
| Pre-test | -0.097 (-0.225-0.046) | -0.073 (-0.349-0.316) | p=0.689 (z=-0.435) |
| Post-test | -0.031 (-0.247-0.066) | 0.039 (-0.159-0.099) | p=0.503 (z=-0.707) |
| Follow-up | -0.097 (-0.352-0.192) | -0.009 (-0.275-0.103) | p=0.769 (z=-0.326) |
|  | p= 0.686 | p= 0.978 |  |

†, significant within-group difference from pretest to follow-up; §, significant within-group difference from pretest to post-test; ‡, significant within-group difference from post-test to follow-up; *, significant between-group difference

Abbreviations: RAeT, resistance+aerobic training; AT, agility training; PFC, prefrontal cortex; PMC, premotor cortex; SMA, supplementary motor area

**Supplementary Table 5. Brain activation indicated by HbO in different channels under Wisconsin card sort test (WCST)**

| **WCST** | AT | RAeT |  |
| --- | --- | --- | --- |
|  | Med (Q1-Q3) | Med (Q1-Q3) |  |
| PFC (ch1: S1D1) | | |  |
| Pre-test | -0.010 (-0.279-0.134) | 0.008 (-0.333-0.224) | p=0.810 |
| Post-test | -0.149 (-0.239-0.043) | 0.081 (-0.260-0.262) | p=0.205 |
| Follow-up | -0.014 (-0.239-0.292) | 0.030 (-0.261-0.211) | p=0.979 |
|  | p= 0.763 | p= 0.978 |  |
| PFC (ch2: S2D2) | | |  |
| Pre-test | -0.024 (-0.230-0.140) | -0.101 (-0.225-0.130) | p=0.936 |
| Post-test | -0.142 (-0.340-0.002) | 0.057 (-0.235-0.428) | p=0.077 |
| Follow-up | -0.093 (-0.444-0.063) | 0.114 (-0.102-0.234) | p=0.225 |
|  | p=0.542 | p=0.191 |  |
| PMC (ch3: S3D3) | | |  |
| Pre-test | 0.082 (-0.292-0.230) | -0.046 (-0.250-0.331) | p=0.852 |
| **Post-test** | **-0.056(-0.393-0.027)*** | **0.268 (-0.086-0.487)*** | **p=0.014** |
| Follow-up | -0.185(-0.253-0.379) | 0.220 (-0.373-0.446) | p=0.538 |
|  | p= 0.393 | p= 0.654 |  |
| PMC (ch4:S3D5) | | |  |
| Pre-test | 0.110 (-0.099-0.249) | -0.006 (-0.274-0.366) | p=0.650 |
| Post-test | -0.072 (-0.120-0.047) | 0.145 (-0.149-0.303) | p=0.052 |
| Follow-up | -0.113 (-0.258-0.029) | 0.151 (-0.473-0.468) | p=0.406 |
|  | p=0.114 | p=0.856 |  |
| PMC (ch7: S5D3) | | |  |
| Pre-test | 0.121 (-0.082-0.400) | -0.034 (-0.152-0.388) | p= 0.650 |
| Post-test | 0.052 (-0.028-0.164) | 0.117 (-0.282-0.478) | p= 0.650 |
| Follow-up | 0.032 (-0.313-0.206) | 0.106 (-0.068-0.371) | p= 0.574 |
|  | p=0.763 | p=0.978 |  |
| PMC (ch8: S5D5) | | |  |
| Pre-test | 0.076 (-0.015-0.372) | 0.049 (-0.067-0.275) | p= 0.538 |
| Post-test | -0.025 (-0.152-0.106) | 0.076 (-0.065-0.438) | p= 0.247 |
| Follow-up | 0.003 (-0.181-0.264) | 0.088 (-0.182-0.497) | p= 0.650 |
|  | p= 0.517 | p= 0.978 |  |
| PMC (ch5: S4D4) | | |  |
| Pre-test | 0.015 (-0.323-0.247) | -0.017 (-0.259-0.058) | p=0.769 |
| Post-test | -0.177 (-0.506-0.090) | 0.013 (-0.330-0.427) | p=0.769 |
| Follow-up | -0.176 (-0.565-0.031) | 0.214 (-0.391-0.428) | p=0.152 |
|  | p= 0.314 | p= 0.191 |  |
| PMC (ch6: S4D6) | | |  |
| Pre-test | 0.019 (-0.225-0.210) | 0.107 (-0.119-0.265) | p= 0.503 |
| Post-test | -0.066 (-0.263-0.123) | 0.100 (-0.179-0.368) | p= 0.320 |
| Follow-up | -0.081 (-0.384-0.058) | 0.136 (-0.175-0.354) | p= 0.168 |
|  | p=0.437 | p=0.654 |  |

| PMC (ch9: S6D4) | | |  |
| --- | --- | --- | --- |
| Pre-test | 0.024 (-0.105-0.233) | 0.010 (-0.129-0.193) | p= 0.769 |
| Post-test | -0.060 (-0.235-0.083) | 0.001 (-0.169-0.282) | p= 0.347 |
| **Follow-up** | **-0.102 (-0.268-0.048)*** | **0.077 (0.018-0.275)*** | **p= 0.011** |
|  | p=0.353 | p=0.108 |  |
| PMC (ch10: S6D6) | | |  |
| Pre-test | 0.005 (-0.242-0.254) | 0.052 (-0.060-0.205) | p= 0.689 |
| Post-test | -0.087 (-0.346-0.002) | 0.038 (-0.185-0.407) | p= 0.152 |
| Follow-up | -0.085 (-0.265-0.125) | 0.099 (-0.345-0.279) | p= 0.650 |
|  | p= 0.314 | p= 0.856 |  |
| SMA (ch11: S7D7) | | |  |
| Pre-test | 0.028 (-0.152-0.171) | 0.017 (-0.237-0.287) | p=0.728 |
| Post-test | -0.072 (-0.243-0.132) | 0.111 (-0.071-0.317) | p=0.087 |
| Follow-up | -0.064 (-0.166-0.050) | 0.125 (-0.285-0.348) | p=0.376 |
|  | p= 0.806 | p= 0.978 |  |
| SMA (ch13: S8D7) | | |  |
| Pre-test | 0.030 (-0.418-0.225) | 0.044 (-0.155-0.258) | p=0.503 |
| Post-test | -0.003 (-0.198-0.106) | 0.355 (-0.023-0.545) | p=0.123 |
| Follow-up | 0.040 (-0.208-0.152) | 0.085 (-0.193-0.180) | p=0.650 |
|  | p=0.897 | p=0.383 |  |
| SMA (ch12: S7D8) | | |  |
| Pre-test | -0.001 (-0.186-0.192) | 0.114 (-0.102-0.218) | p=0.247 |
| Post-test | -0.048 (-0.203-0.036) | 0.142 (-0.127-0.402) | p=0.087 |
| Follow-up | -0.054 (-0.192-0.078) | 0.142 (-0.207-0.373) | p=0.123 |
|  | p=0.429 | p=0.751 |  |
| SMA (ch14: S8D8) | | |  |
| Pre-test | 0.104 (-0.250-0.268) | 0.010 (-0.143-0.298) | p=0.979 |
| Post-test | -0.022 (-0.142-0.137) | 0.179 (-0.067-0.306) | p=0.098 |
| Follow-up | -0.024 (-0.140-0.141) | 0.056 (-0.405-0.149) | p=0.894 |
|  | p=0.925 | p=0.434 |  |

†, significant within-group difference from pretest to follow-up; §, significant within-group difference from pretest to post-test; ‡, significant within-group difference from post-test to follow-up; *, significant between-group difference

Abbreviations: RAeT, resistance+aerobic training; AT, agility training; PFC, prefrontal cortex; PMC, premotor cortex; SMA, supplementary motor area

**Supplementary Table 6. Brain activation indicated by HbO in different channels under single-task walking**

| **Single-task walking** | **AT** | **RAeT** |  |
| --- | --- | --- | --- |
|  | **Med (Q1-Q3)** | **Med (Q1-Q3)** |  |
|  | | |  |
| PFC (ch1: S1D1) | | |  |
| Pre-test | -0.312 (-0.498-0.203) | -0.551 (-0.956- -0.180) | p=0.123 |
| Post-test | -0.505 (-0.839-0.128) | -0.551 (-0.941- -0.067) | p=0.689 |
| Follow-up | -0.150 (-0.869-0.026) | -0.700 (-1.026- -0.348) | p=0.137 |
|  | p= 0.245 | p= 0.285 |  |
| PFC (ch2: S2D2) | | |  |
| Pre-test | -0.237 (-0.488- -0.115) | -0.515 (-0.836- -0.048) | p=0.277 |
| Post-test | -0.570 (-0.706-0.033) | -0.515 (-0.724- -0.145) | p=0.910 |
| Follow-up | -0.266 (-0.516-0.064) | -0.437 (-1.054- -0.222) | p=0.150 |
|  | p=0.304 | p=0.994 |  |
| PMC (ch3: S3D3) | | |  |
| Pre-test | -0.172 (-0.682-0.427) | -0.574 (-1.047-0.369) | p= 0.574 |
| Post-test | -0.155 (-0.573-0.371) | -0.451 (-1.043-0.144) | p= 0.247 |
| Follow-up | -0.309 (-0.785-0.255) | -0.449 (-1.105- -0.326) | p= 0.270 |
|  | p= 0.955 | p= 0.367 |  |
| PMC (ch4: S3D5) | | |  |
| Pre-test | -0.305 (-1.015-0.096) | -0.319 (-0.727- -0.061) | p= 0.852 |
| Post-test | -0.264 (-0.582- -0.075) | -0.388 (-0.709-0.236) | p= 0.852 |
| Follow-up | -0.270 (-0.676- -0.059) | -0.399 (-0.918- -0.176) | p= 0.574 |
|  | p= 0.995 | p= 0.974 |  |
| PMC (ch7: S5D3) | | |  |
| Pre-test | 0.156 (-0.175-0.282) | -0.213 (-0.669-0.066) | p= 0.270 |
| Post-test | -0.027 (-0.331-0.166) | -0.203 (-0.769-0.390) | p= 0.650 |
| Follow-up | 0.168 (-0.330-0.308) | -0.091 (-0.603-0.164) | p= 0.225 |
|  | p= 0.783 | p= 0.556 |  |
| PMC (ch8: S5D5) | | |  |
| Pre-test | -0.089 (-0.442-0.315) | -0.342 (-0.670- -0.187) | p= 0.168 |
| Post-test | -0.265 (-0.394- -0.038) | -0.342 (-0.900-0.087) | p= 0.728 |
| Follow-up | -0.166 (-0.570-0.047) | -0.253 (-0.863-0.309) | p= 0.728 |
|  | p= 0.931 | p= 0.636 |  |
| PMC (ch5: S4D4) | | |  |
| Pre-test | -0.344 (-0.863- -0.099) | -0.279 (-0.745- -0.072) | p=0.769 |
| Post-test | -0.408 (-1.109- -0.138) | -0.283 (-1.509- -0.102) | p=0.979 |
| Follow-up | -0.429 (-0.572-0.031) | -0.567 (-0.849- -0.066) | p=0.503 |
|  | p= 0.464 | p= 0.318 |  |
| PMC (ch6: S4D6) | | |  |
| Pre-test | -0.240 (-0.704-0.006) | -0.463 (-0.876- -0.320) | p= 0.152 |
| Post-test | -0.292 (-0.544- -0.127) | -0.406 (-0.903- -0.255) | p= 0.225 |
| Follow-up | -0.242 (-0.661- -0.020) | -0.406 (-0.657- -0.271) | p= 0.611 |
|  | p= 0.710 | p= 0.761 |  |
| PMC (ch9: S6D4) | | |  |
| Pre-test | -0.203 (-0.278-0.073) | -0.108 (-0.600-0.44) | p= 0.852 |
| Post-test | -0.247 (-0.601-0.137) | -0.442 (-0.897-0.040) | p= 0.376 |
| Follow-up | -0.258 (-0.387- -0.110) | -0.249 (-0.687-0.043) | p= 0.894 |
|  | p= 0.798 | p= 0.234 |  |
| PMC (ch10: S6D6) | | |  |
| Pre-test | -0.226 (-0.509-0.029) | -0.201 (-0.962-0.130) | p= 0.894 |
| Post-test | -0.255 (-0.948-0.001) | -0.634 (-1.186-0.154) | p= 0.936 |
| Follow-up | -0.367 (-0.715-0.051) | -0.678 (-1.591-0.025) | p= 0.406 |
|  | p= 0.320 | p= 0.452 |  |
| SMA (ch11: S7D7) | | |  |
| Pre-test | -0.385 (-0.642- -0.149) | -0.667 (-0.873-0.055) | p=0.611 |
| Post-test | -0.311 (-0.805- -0.126) | -0.388 (-0.679-0.044) | p=0.769 |
| Follow-up | -0.292 (-0.832- -0.163) | -0.667 (-1.261- -0.125) | p=0.538 |
|  | p= 0.574 | p= 0.156 |  |
| SMA (ch13: S8D7) | | |  |
| Pre-test | -0.316 (-0.567- -0.090) | -0.233 (-0.868-0.116) | p=0.936 |
| Post-test | -0.364 (-0.686- -0.273) | -0.628 (-0.981-0.078) | p=0.728 |
| Follow-up | -0.366 (-0.657- -0.247) | -0.431 (-0.870-0.078) | p=0.769 |
|  | p=0.608 | p=0.802 |  |
| SMA (ch12: S7D8) | | |  |
| Pre-test | -0.338 (-0.639- -0.087) | -0.530 (-0.705- -0.271) | p=0.470 |
| Post-test | -0.631 (-0.930- -0.232) | -0.539 (-0.670- -0.271) | p=0.689 |
| Follow-up | -0.597 (-0.803- -0.037) | -0.577 (-1.137- -0.346) | p=0.574 |
|  | p= 0.177 | p= 0.256 |  |
| SMA (ch14: S8D8) | | |  |
| Pre-test | -0.162 (-0.446- -0.109) | -0.501 (-0.691-0.121) | p= 0.347 |
| Post-test | -0.261 (-0.479-0.176) | -0.287 (-0.724- -0.131) | p= 0.260 |
| Follow-up | -0.266 (-0.481-0.207) | -0.354 (-0.899- -0.126) | p= 0.449 |
|  | p= 0.801 | p= 0.868 |  |

†, significant within-group difference from pretest to follow-up; §, significant within-group difference from pretest to post-test; ‡, significant within-group difference from post-test to follow-up; *, significant between-group difference

Abbreviations: RAeT, resistance+aerobic training; AT, agility training; PFC, prefrontal cortex; PMC, premotor cortex; SMA, supplementary motor area

**Supplementary Table 7. Brain activation indicated by HbO in different channels under dual-task walking**

| **Dual-task walking** | **AT** | **RAeT** |  |
| --- | --- | --- | --- |
|  | **Med (Q1-Q3)** | **Med (Q1-Q3)** |  |
| PFC (ch1: S1D1) | | |  |
| Pre-test | -0.091 (-0.497-0.191) | -0.256 (-0.790-0.107) | p= 0.538 |
| **Post-test** | **-0.091 (-0.308-0.038)*** | **-0.621 (-0.898-0.080)*** | **p=0.046** |
| Follow-up | -0.191 (-0.488-0.159) | -0.552 (-1.039-0.003) | p=0.087 |
|  | p= 0.625 | p=0.346 |  |
| PFC (ch2: S2D2) | | |  |
| Pre-test | -0.081 (-0.502-0.563) | -0.274 (-0.794-0.558) | p=0.424 |
| Post-test | -0.081 (-0.502-0.499) | -0.578 (-0.997-0.016) | p=0.106 |
| Follow-up | -0.257 (-0.356-0.058) | -0.560 (-0.926-0.018) | p=0.252 |
|  | p= 0.464 | p= 0.880 |  |
| PMC (ch3: S3D3) | | |  |
| Pre-test | -0.157 (-0.803-0.389) | 0.063 (-0.729-0.461) | p=0.470 |
| Post-test | -0.202 (-0.703-0.890) | -0.352 (-0.922-0.237) | p=0.434 |
| Follow-up | 0.169 (-0.812-0.918) | -0.520 (-1.136-0.161) | p=0.123 |
|  | p=0.330 | p=0.075 |  |
| PMC (ch4: S3D5) | | |  |
| Pre-test | -0.123 (-0.558-0.231) | -0.221 (-0.802-0.128) | p=0.728 |
| Post-test | -0.266 (-0.576-0.338) | -0.529 (-1.006- -0.209) | p=0.205 |
| Follow-up | -0.219 (-0.578-0.239) | -0.367 (-0.997- -0.090) | p=0.152 |
|  | p= 0.552 | p= 0.369 |  |
| PMC (ch7: S5D3) | | |  |
| Pre-test | 0.411 (0.015-0.601) | 0.208 (-0.243-0.724) | p= 0.320 |
| Post-test | 0.197 (-0.172-0.562) | -0.064 (-0.491-0.673) | p= 0.406 |
| Follow-up | 0.458 (0.048-0.551) | 0.072 (-0.649-0.669) | p= 0.168 |
|  | p= 0.696 | p= 0.234 |  |
| PMC (ch8: S5D5) | | |  |
| Pre-test | 0.207 (-0.647-1.010) | -0.145 (-0.558-0.479) | p= 0.769 |
| Post-test | -0.194 (-0.647-0.577) | -0.353 (-0.831-0.572) | p= 0.538 |
| Follow-up | -0.048 (-0.337-0.827) | -0.194 (-0.975-0.500) | p= 0.295 |
|  | p= 0.356 | p= 0.253 |  |
| PMC (ch5: S4D4) | | |  |
| Pre-test | -0.169 (-0.731- -0.045) | -0.164 (-0.535-0.448) | p=0.437 |
| Post-test | -0.342 (-1.021- -0.073) | -0.519 (-1.172-0.498) | p=0.574 |
| Follow-up | -0.305 (-0.724-0.381) | -0.419 (-1.149-0.351) | p=0.437 |
|  | p= 0.710 | p= 0.317 |  |
| PMC (ch6: S4D6) | | |  |
| Pre-test | -0.218 (-0.419-0.043) | -0.471 (-0.661-0.074) | p=0.347 |
| Post-test | -0.021 (-0.625-0.150) | -0.513 (-0.908- -0.136) | p=0.168 |
| Follow-up | -0.199 (-0.709-0.243) | -0.495 (-1.145-0.008) | p=0.186 |
|  | p= 0.710 | p= 0.426 |  |

| PMC (ch9: S6D4) | | |  |
| --- | --- | --- | --- |
| Pre-test | 0.134 (-0.400-0.274) | -0.129 (-0.350-0.548) | p= 0.852 |
| Post-test | -0.116 (-0.747-0.271) | -0.375 (-0.914-0.125) | p= 0.406 |
| Follow-up | -0.159 (-0.709-0.688) | -0.440 (-0.855-0.107) | p= 0.168 |
|  | p= 0.464 | p= 0.103 |  |
| PMC (ch10: S6D6) | | |  |
| Pre-test | 0.019 (-0.788-0.450) | **-0.079 (-0.407-0.423)** | p= 0.810 |
| Post-test | -0.205 (-0.863-0.240) | **-0.357 (-0.610-0.012)** **§** | p= 0.728 |
| Follow-up | -0.258 (-0.878-0.606) | **-0.539 (-1.264- -0.202)** | p= 0.152 |
|  | p= 0.666 | **p= 0.048 (pre-post: 0.016,** post-f/u: 0.052, pre-f/u: 0.424) |  |
| SMA (ch11: S7D7) | | |  |
| Pre-test | -0.418 (-0.725- -0.026) | -0.256 (-0.703-0.154) | p=0.147 |
| Post-test | -0.563 (-0.810- -0.197) | -0.752 (-0.959- -0.067) | p=0.538 |
| Follow-up | -0.501 (-0.815- -0.063) | -0.838 (-1.228- -0.264) | p=0.376 |
|  | p= 0.888 | p= 0.055 |  |
| SMA (ch13: S8D7) | | |  |
| Pre-test | -0.162 (-0.820-0.091) | -0.154 (-0.442-0.243) | p= 0.406 |
| Post-test | -0.161 (-0.506-0.064) | -0.492 (-0.737-0.001) | p= 0.406 |
| Follow-up | -0.353 (-0.735- -0.003) | -0.412 (-0.695-0.240) | p= 0.979 |
|  | p= 0.660 | p= 0.136 |  |
| SMA (ch12: S7D8) | | |  |
| Pre-test | -0.355 (-0.666- -0.036) | -0.405 (-0.640- -0.012) | p=0.979 |
| Post-test | -0.486 (-0.866-0.208) | -0.637 (-0.931- -0.420) | p=0.320 |
| Follow-up | -0.408 (-0.634- -0.014) | -0.754 (-1.237- -0.128) | p=0.295 |
|  | p= 0.615 | p= 0.630 |  |
| SMA (ch14: S8D8) | | |  |
| Pre-test | -0.155 (-0.288-0.076) | -0.208 (-0.337- -0.019) | p=0.650 |
| Post-test | -0.129 (-0.691-0.234) | -0.333 (-0.522- -0.021) | p=0.538 |
| Follow-up | -0.260 (-0.779- -0.010) | -0.196 (-0.687- 0.280) | p=0.650 |
|  | p= 0.541 | p= 0.754 |  |

†, significant within-group difference from pretest to follow-up; §, significant within-group difference from pretest to post-test; ‡, significant within-group difference from post-test to follow-up; *, significant between-group difference

Abbreviations: RAeT, resistance+aerobic training; AT, agility training; PFC, prefrontal cortex; PMC, premotor cortex; SMA, supplementary motor area

# Supplementary Figures

**Supplementary Figure 1. Hurdle drill (fencing mask is hurdles). Double-legged jump and single-legged jump**


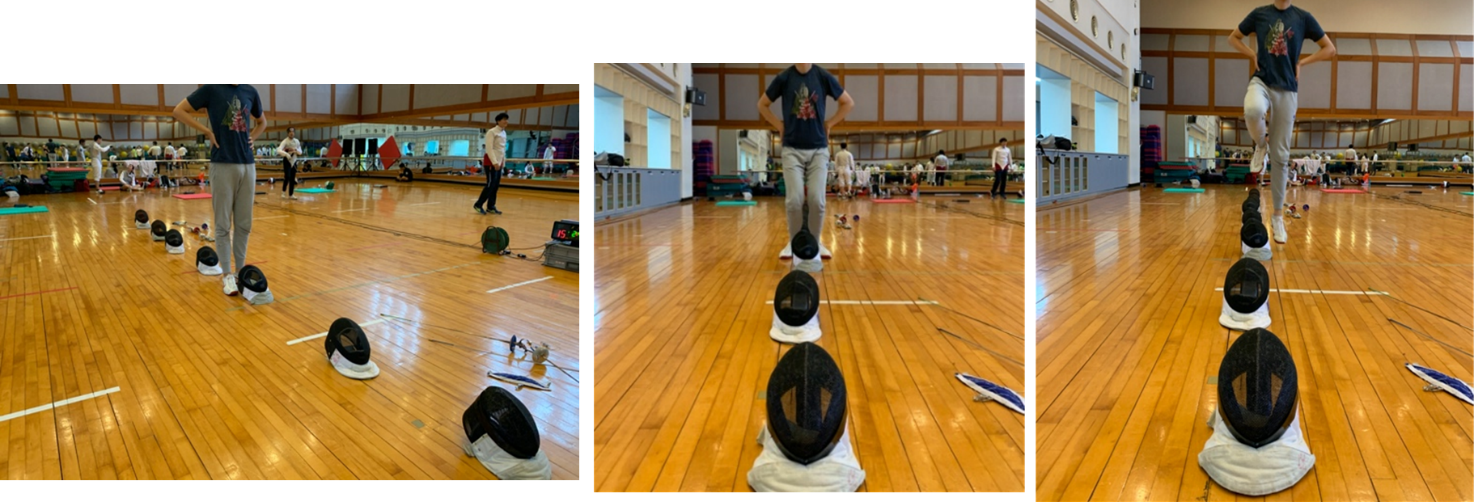


**Supplementary Figure 2. Plyometric box drill. Double-legged jump and single-legged jump.**


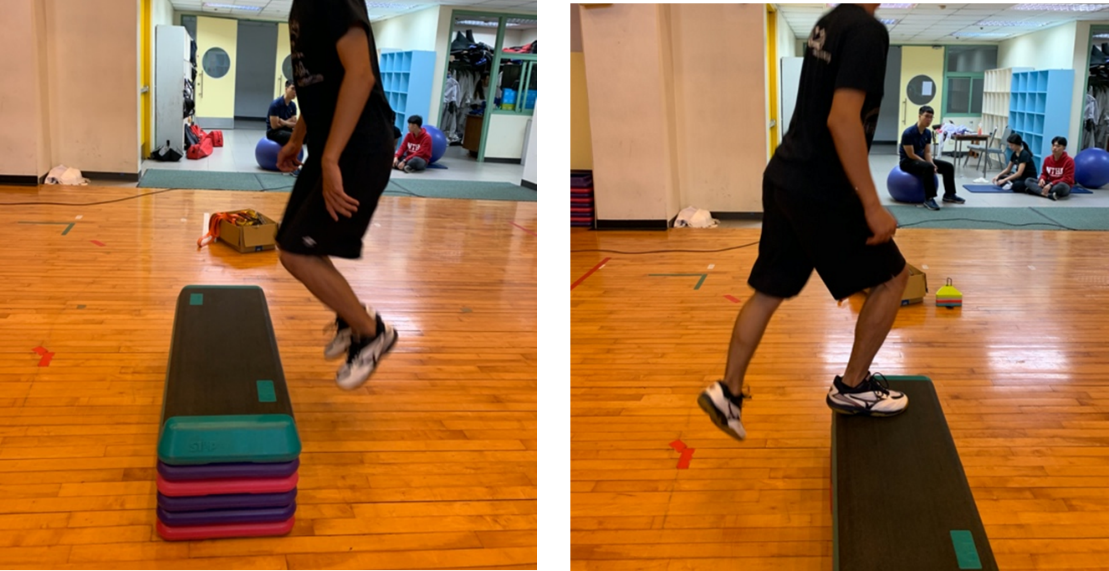


**Supplementary Figure 3. Cone drill. Touch the cone or bypass the cone**

**
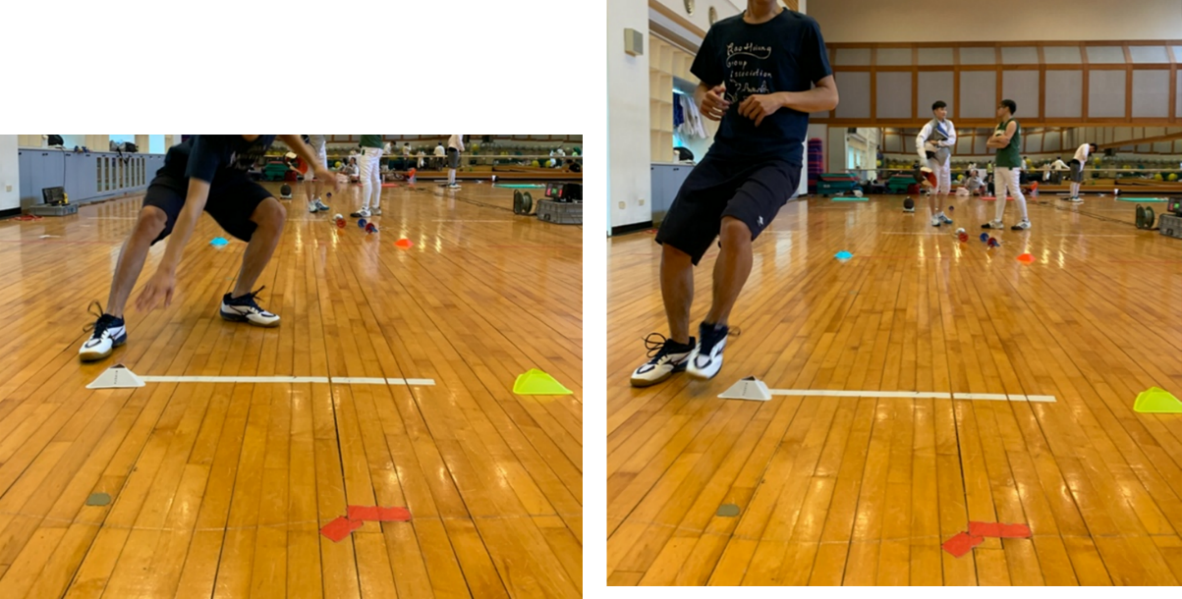
**

**Supplementary Figure 4. Agility ball drill. Throw the ball to the ground and then catch it by one hand while running. Both hands take turns.**

**
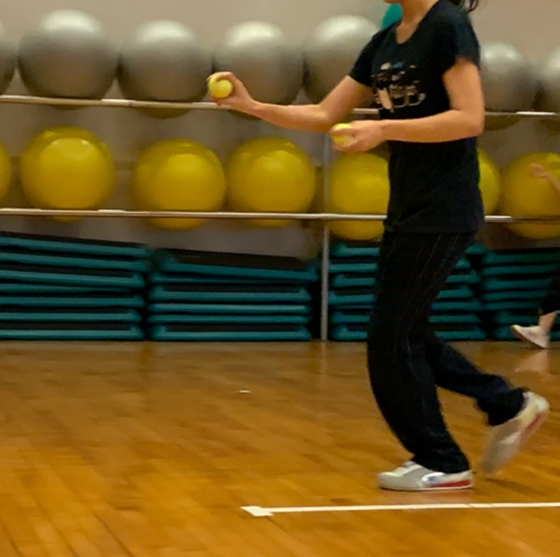
**

**Supplementary Figure 5. Mirror drill. One person imitates the other’s action and direction**

**
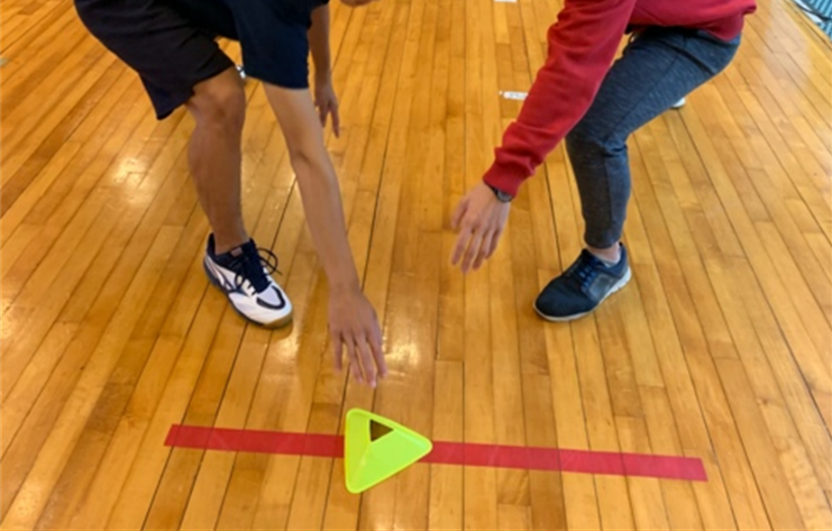
**
